# Diversity of Nuclear Lamin A/C Action as a Key to Tissue-Specific Regulation of Cellular Identity in Health and Disease

**DOI:** 10.3389/fcell.2021.761469

**Published:** 2021-10-13

**Authors:** Anna Malashicheva, Kseniya Perepelina

**Affiliations:** Laboratory of Regenerative Biomedicine, Institute of Cytology, Russian Academy of Sciences, St. Petersburg, Russia

**Keywords:** lamin A/C, laminopathies, *LMNA* gene, cell differentiation, LADs, regulation of gene expression, chromatin organization

## Abstract

A-type lamins are the main structural components of the nucleus, which are mainly localized at the nucleus periphery. First of all, A-type lamins, together with B-type lamins and proteins of the inner nuclear membrane, form a stiff structure—the nuclear lamina. Besides maintaining the nucleus cell shape, A-type lamins play a critical role in many cellular events, such as gene transcription and epigenetic regulation. Nowadays it is clear that lamins play a very important role in determining cell fate decisions. Various mutations in genes encoding A-type lamins lead to damages of different types of tissues in humans, collectively known as laminopathies, and it is clear that A-type lamins are involved in the regulation of cell differentiation and stemness. However, the mechanisms of this regulation remain unclear. In this review, we discuss how A-type lamins can execute their regulatory role in determining the differentiation status of a cell. We have summarized recent data focused on lamin A/C action mechanisms in regulation of cell differentiation and identity development of stem cells of different origin. We also discuss how this knowledge can promote further research toward a deeper understanding of the role of lamin A/C mutations in laminopathies.

## Introduction

A-type lamins are the structural components of the nucleus, which together with B-type lamins and inner nuclear membrane proteins form a scaffold, termed the nuclear lamina. Mostly A-type lamins are included in the lamina at the nucleus periphery; however, a small fraction of lamin A/C is found throughout the nucleoplasm (Naetar et al., 2017; Briand and Collas, 2020). Primarily, nuclear lamin A/C was thought to undertake solely a structural role, providing shape and stiffness to the nucleus. Currently, A-type lamins are known as essential regulators of gene expression and key mediators of cell fate determination. Lamin A/C participation in chromatin organization, DNA replication, gene transcription regulation, cell differentiation, and tissue-specific functions has been extensively investigated (de Leeuw et al., 2018; Zhang et al., 2019; Alcorta-Sevillano et al., 2020). A-type lamins perform most of these functions by interacting with the inner nuclear membrane proteins, transcription factors, and DNA (Parnaik, 2008; Prokocimer et al., 2009; Lambert, 2019; Almendáriz-Palacios et al., 2020). A-type lamins are believed to regulate important signaling pathways’ activity in cells (such as Rb/E2F, Wnt/β-catenin, TGFβ, Notch) through their direct or indirect interactions with other proteins (Maraldi et al., 2010, 2011; Gerace and Tapia, 2018).

In this review we mainly focused on recent data about the role of lamin A/C in cell differentiation. The latter has become a focus of increasing attention over the past decade, initially because of the identification of new mutations in the *LMNA* gene and associated diseases—laminopathies (Wong and Stewart, 2020). The most famous lamin modification is progerin, causing a severe developmental disorder—premature aging syndrome, or progeria. This known disease is extremely rare (Worman, 2012; Schreiber and Kennedy, 2013; Gonzalo et al., 2017). At the same time, *LMNA* point mutations leading to damage of various types of tissues occur more often. It is supposed that the development of the disease is associated with an abnormality of the stem cell differentiation process. Taking into account that A-type lamins are expressed in all differentiated cell types, it seems still unclear why only certain differentiated tissues are selectively affected for each type of laminopathy (Worman, 2012; Gruenbaum and Foisner, 2015; Robson et al., 2016; Zhang et al., 2019).

Recently, research has focused on the molecular mechanisms of laminopathies’ development. The mechanisms proposed for the pathology development include disturbances in chromatin organization, intracellular signal transduction, as well as epigenetic changes. Consequently, all this leads to dysregulation of genes responsible for cell differentiation (van Steensel and Belmont, 2017; Zhang et al., 2019). Regions of lamin–chromatin interaction (lamina-associated domains—LADs) are known to be implicated in regulation of gene expression. LADs contain a diversity of differentiation-related genes, that are in an active or inactive state depending on their association with chromatin. Active gene expression is associated with releasing LADs from the nuclear lamina. In contrast, inactivation of expression is associated with the attachment of LADs to the lamina (Briand and Collas, 2020; Bitman-Lotan and Orian, 2021; Shah et al., 2021).

Apparently, cellular context is essential for further development of tissue-specific disease phenotypes. There is strong evidence of lamin A/C involvement in the processes of cell differentiation, in particular, in the adipogenic (Oldenburg et al., 2017; Perepelina et al., 2018), osteogenic (Avnet et al., 2011; Alcorta-Sevillano et al., 2020), myogenic (Steele-Stallard et al., 2018), and cardiogenic (Mounkes et al., 2005; Shah et al., 2021) directions. Recently, a theory has been proposed about the action of mechanical signals coming from the extracellular matrix to the lamin network resulting in a redistribution of chromatin and a change in the availability of DNA for transcription factors (Osmanagic-Myers et al., 2015; Martino et al., 2018; Donnaloja et al., 2020). Recent studies show that all these events affect the biophysical properties of the nucleus, which, in turn, affects the fate and differentiation of cells (Alcorta-Sevillano et al., 2020).

The difficulties in studying the role of lamin A/C in cell differentiation are in particular related to the lack of a unified experimental model. Some studies were performed using mesenchymal stem cells, fibroblast (van Tienen et al., 2019; Ikegami et al., 2020), cardiac mesenchymal cells (Perepelina et al., 2019), osteoblast precursors (Avnet et al., 2011; Y. Liu et al., 2012), etc. Some studies used mouse models (Arimura et al., 2005; Mounkes et al., 2005; Le Dour et al., 2017; Hamczyk et al., 2018). Currently, studying the mechanisms of laminopathies’ development using induced pluripotent stem cell (iPSC) models is especially relevant (Crasto and Di Pasquale, 2018; Steele-Stallard et al., 2018; Giacomelli et al., 2020; Shah et al., 2021).

Here we summarized the current knowledge about the crucial role of lamin A/C in regulating differentiation of stem cells of various origin. We specifically focus on the issue of how the regulation of differentiation by A-type lamins could be involved in the pathogenesis of laminopathies for which generally accepted concept of the pathogenesis is still absent.

## Organization, Maturation, and Assembly of Lamin A/C

**FIGURE 1:**
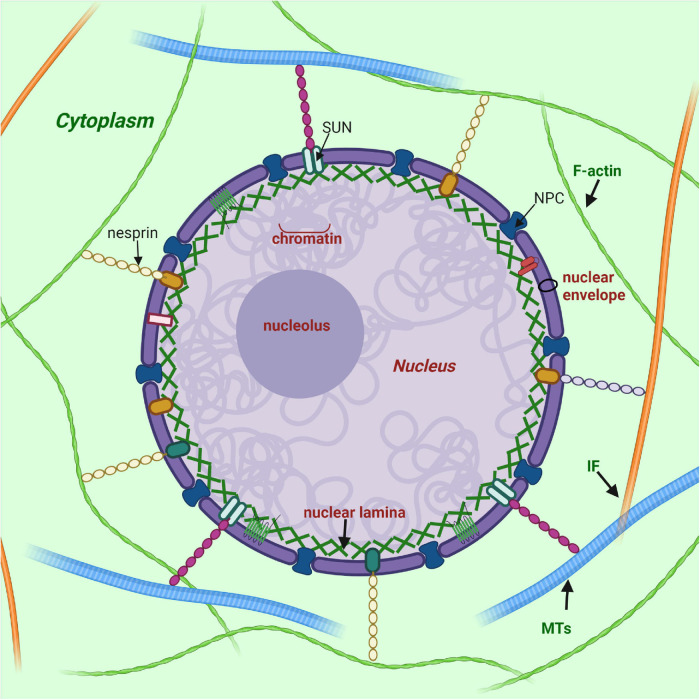
Nuclear lamina position and its interplay with other structures of cell. Nuclear lamina is a stiff meshwork consisting of A-type lamins and B-type localized between the nuclear envelope and chromatin. Nuclear lamins interact with a wide range of nuclear envelope proteins (NEPs). Also, nuclear lamins can interact with the cytoskeleton (filamentous actin – F actin; microtubules – MTs; and intermediate filaments – IF) *via* SUN proteins and nesprins. Created with BioRender.com.

Nuclear lamins in metazoan cells are members of the type V intermediate filament (IF) family. There are two groups of lamins, the A type and the B type, which, in association with inner nuclear membrane proteins, form a stiff meshwork under the inner nuclear membrane termed the nuclear lamina (Figure 1). While B-type lamins are expressed overall in all cells, A-type lamins are only expressed in differentiated cells, which apparently determines the specific functions of this type of lamin in the cell. Moreover, the expression level of lamin A/C varies in different tissues (Gruenbaum and Foisner, 2015). As a result of alternative splicing of *LMNA* gene transcript, several isoforms such as A, C and minor isoforms AΔ10 and C2 are generated.

**FIGURE 2:**
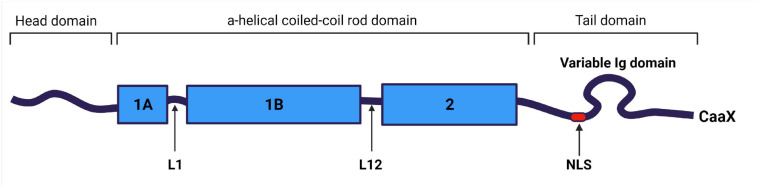
Structural organization of lamin A/C filament. Lamin A/C is an intermediate filament, and contains a central coiled-coil rod domain divided into 1A, 1B, and 2 sub-domains connected *via* L1 and L12 linkers; a head domain; and a tail domain containing nuclear localization signal (NLS), Ig-like domain and carboxyterminal CaaX box (apart of lamin C), where C is cysteine, a – aliphatic amino acid, and X can be any amino acid. Created with BioRender.com.

Along with all IFs, lamin filaments contain three structural domains: a central α-helical rod domain, a short globular amino-terminal “head” domain and a long carboxyterminal “tail” domain. The rod domain of lamins includes three helical segments (1A, 1B, and 2), connected by short linkers L1 and L12 (Ahn et al., 2019). Lamins have several differences from cytoplasmic IFs: (1) they contain 42 additional amino acids in their rod domain; (2) they have a shorter head domain; and (3) their carboxyl-terminal “tail” domain includes the nuclear localization signal (NLS)—which is required for their nuclear transport after synthesis in the cytoplasm—, an immunoglobulin-like (Ig-) fold domain, a chromatin binding site, and—with the exception of lamin C—a CaaX motif (where C is cysteine, a—an aliphatic amino acid, and X—any amino acid) (Wu et al., 2014; Gruenbaum and Foisner, 2015; Figure 2).

**FIGURE 3:**
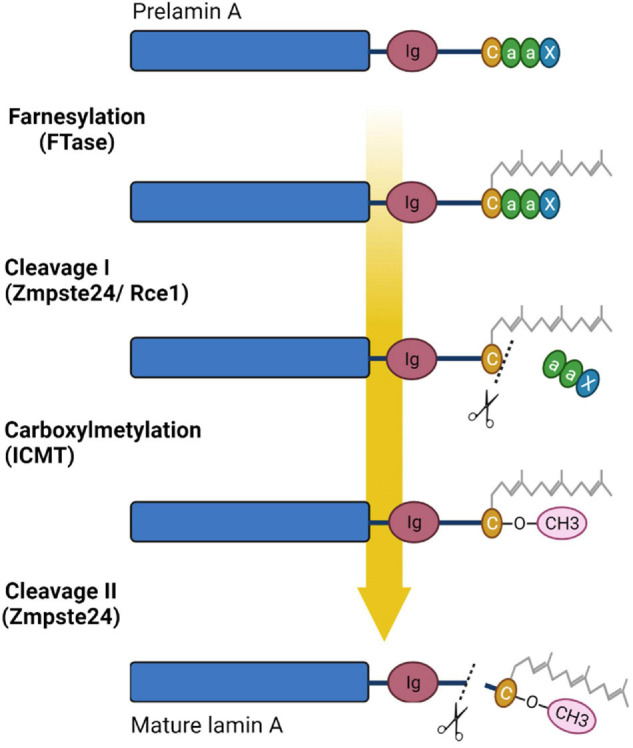
Processing of prelamin A. Prelamin A undergoes four steps of reaction to become mature lamin A: farnesylation of cysteine of CaaX box by farnesyltransferase enzyme (FTase), cleavage of aaX by the zinc metalloprotease Zmpste24 (FACE1) or RAS converting enzyme 1 (Rce1), carboxylmethylation of the farnesylated cysteine *via* the isoprenylcysteine carboxyl methyltransferase (ICMT), and finally cleavage of the 15 terminal amino acids, including the farnesylated and carboxymethylated cysteine, by ZMPSTE24. Created with BioRender.com.

Lamin C is translated as a mature protein without multiple post-translated modifications as in the case of A-type lamin, and lacking 98 amino acids with CaaX motif; A-type lamin is expressed in cells as prelamin, which undergoes multiple post-translational modifications of the carboxyterminal “tail” domain. At the first stage of prelamin A processing, farnesyltransferase enzyme (FTase) adds a farnesyl group to the C-terminal cysteine. Then the three residues (aaX) are cleavaged *via* the zinc metalloprotease Zmpste24 (FACE1) or RAS converting enzyme 1 (Rce1). At the next stage C-terminal cysteine is carboxymethylated by the isoprenylcysteine carboxyl methyltransferase (ICMT). Finally, enzyme Zmpste24 cleaves the last 15 C-terminal amino-acids of lamin A, thereby removing the carboxy farnesylated and methylated cysteine (Fisher et al., 1986; Corrigan et al., 2005; Adam et al., 2013; Wu et al., 2014). Figure 3 presents the lamin A post-translational modifications during maturation.

Other types of post-translational modifications of lamins are known such as sumoylation, ubiquitylation, acetylation, and phosphorylation. These modifications obviously play a significant role in regulating lamin translocation during the cell cycle (Prokocimer et al., 2009; Donnaloja et al., 2020). Phosphorylation of lamins is involved in plenty of cellular process. To date, some research has shown that phosphorylation contributes to the interaction between B-type lamins and histone H2A/H2B in *Drosophila* (Mattout et al., 2007). In mammalian cells two specific sites flanking the lamin’s rod domain are phosphorylated by cyclin-dependent kinase (CDK)-1. This event is required for lamin disassembly into dimers during mitosis (Chaffee et al., 2014; Naetar et al., 2017). Moreover, phosphorylation contributes to the dynamic interaction of lamins with other proteins as well as lamin A/C solubility, and lamina meshwork formation. Remarkably, all these processes could be activated/inactivated, as phosphorylation is a reversible modification (Kochin et al., 2014; Liu and Ikegami, 2020). Apparently, lamin phosphorylation takes place in the modulation of enhancer activity. It has been reported that S22-phosphorylated lamins connect with active genomic enhancer sites and this interaction is violated in progeroid cells (Ikegami et al., 2020). Sumoylation has been shown to be important for normal lamin A/C functions and also for the regulation of lamin A/C assembly (Kim et al., 2011). It has been shown that mutant lamin A/C (E203G and E203K) leads to a decreased level of lamin sumoylation in fibroblasts and increased cell death (Zhang and Sarge, 2008).

**FIGURE 4:**
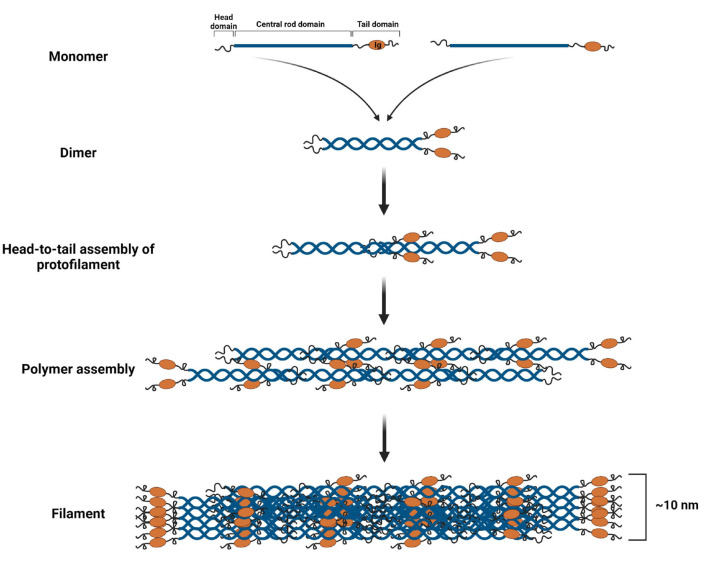
Lamin A/C filament assembly. Lamin A/C dimers are formed from monomers that associate in a parallel, forming a coiled-coil through the central α-helical rod domain. Then lamin A/C dimers are assembled in a head-to-tail manner forming protofilament. Protofilaments through the anti-parallel association of lamin A/C polymers produce a polymer. Finally, several polymers cooperate resulting in a 10 nm lamin A/C filament. Created with BioRender.com.

The main structural unit of lamin A/C filaments is a coiled dimer formed as a result of the interaction of two central rod domains of lamin proteins. These dimers are connected head-to-tail and form protofilaments, which could be combined in various configurations to form 10 nm lamin filaments (Herrmann and Aebi, 2004; Prokocimer et al., 2009; Gruenbaum and Foisner, 2015). The structure of the lamin “tail” domain is similar to immunoglobulin and could mediate specific intermolecular interactions with other proteins (Donnaloja et al., 2020; Figure 4).

Recent evidence suggests that the structure of the nuclear lamina in somatic cells is more complex and less homogeneous than was previously thought. It has been shown by cryo-electron tomography tests that lamin filaments are assembled not into 10 nm fibers, but into 3.5 nm fibers with a protruding zone of Ig domain (Donnaloja et al., 2020). In addition, the presence of intermediate “bridges” between neighboring lamina filaments, the nature of which is unknown, has been shown (Burke and Stewart, 2013). In mammals, most lamins can interact with each other; however, some evidence suggests that the bond strength between different lamins can vary, and that A-type and B-type lamins predominantly polymerize into separate homopolymers.

## Lamin A/C-Binding Proteins

Undoubtedly, A-type lamins are essential components of the nucleus which perform a multiplicity of vital cell functions, from stabilization of nucleus shape to involvement in more complex processes such as cell proliferation, migration, signaling transduction, cell differentiation, and others (Gruenbaum and Foisner, 2015; Enyedi and Niethammer, 2017; Naetar et al., 2017; Karoutas and Akhtar, 2021). The abundance of lamin A/C functions is implemented through their direct or mediated interaction with a plethora of inner nuclear membrane (INM) and nucleoplasm proteins. Over 80 nuclear envelope transmembrane proteins (NETs) have been identified as likely interacting with lamins, by means of proteomic analyses in rat (Schirmer et al., 2003). The most important and widespread NETs include lamina-associated polypeptide (LAP) 1, LAP2a, LMNB receptor (LBR), and emerin (Almendáriz-Palacios et al., 2020). In addition, NETs were shown to vary in different tissues, and could contribute to the tissue-specific lamin A/C actions (Korfali et al., 2012).

Lamin-binding proteins are divided into three general groups: (1) proteins providing mechanical support of the nucleus by interacting with subnuclear elements, chromatin and INM; (2) signaling transmission components taking part in cell regulation of vital processes, such as cell differentiation, homeostasis, etc.; (3) proteins regulating gene expression and chromatin organization (Gruenbaum and Foisner, 2015; Martino et al., 2018; Zhang et al., 2019).

**FIGURE 5:**
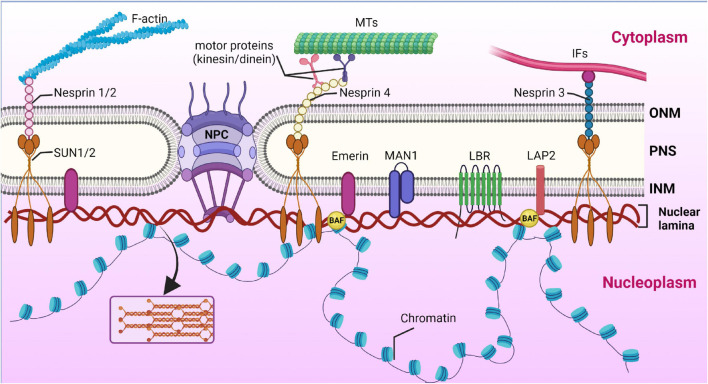
Cooperation of nuclear lamina with nuclear envelope proteins and chromatin. Nuclear lamina is localized between the inner nuclear membrane (INM) and chromatin. Schematic representation of lamin interaction with inner nuclear membrane proteins, the most important of which are MAN1, LAP2, SUN1/2, Emerin, and LBR. The nuclear pore complex (NPC) spans both the inner nuclear membrane (INM) and the outer nuclear membrane (ONM) and mediates macromolecular transport. *Via* SUN1/2 and the nesprins interacting with them, located in the ONM, lamins cooperate with cytoskeleton components, namely filamentous actin (F-actin), microtubules (MTs), and intermediate filaments (IFs). The space between the ONM and INM is termed the perinuclear space (PNS). Created with BioRender.com.

Among the proteins interacting with lamin A/C, emerin and lamina-associated polypeptides (LAPs) are to stand separately. Emerin, LAP2, and MAN1 (called LEM proteins) contain a special domain of 40 amino acid residues termed the LEM domain, which interacts with the barrier to autointegration factor (BAF), a DNA-binding factor involved in organizing chromatin structure and assembling the nuclear envelope. There are LEM proteins that lack a transmembrane domain and therefore they are localized to the nucleoplasm or cytoplasm (Brachner and Foisner, 2011). In addition to the BAF-mediated effect on chromatin structure, lamins interact with epigenetic regulator ING, which binds to core histones, deacetylases, and histone acetyltransferases, as well as mediators of epigenetic regulation. Moreover, lamin A/C can also directly cooperate with chromatin by tethering specific chromatin regions called lamina-associated domains (LADs) at the nuclear periphery (Shevelyov and Ulianov, 2019; Figure 5).

SUN and KASH domain proteins are important nuclear membrane proteins localized in the INM and ONM (outer nuclear membrane), respectively. The SUN proteins interact directly with lamin A/C. KASH proteins bind to major cytoskeleton members, including actin filaments (through nesprin-1 and -2), intermediate filaments (*via* interaction with nesprin-3), and microtubules (*via* kinesin and dynein motor proteins binding to nesprin-1, -2, -4, and KASH5) (Haque et al., 2006). Thus, SUN and KASH domain proteins, nesprins, together with lamin A/C, form a protein complex called the LINC (linker of nucleoskeleton and cytoskeleton) complex, which unites the nucleus and the cytoskeleton and enables force transmission across the nuclear envelope during nuclear positioning and migration (Lee and Burke, 2018).

In addition, through protein–protein interactions, A-type lamins are believed to interact with and regulate the activity and availability of important signaling pathway proteins in the cell, such as Rb/E2F, Wnt/β-catenin, TGFβ, SMAD, and MAPK (Gerbino et al., 2018; Worman, 2018). More details on the participation of lamin A/C and associated proteins will be outlined below.

## Lamin A/C as Tissue-Specific Regulator of Cell Differentiation

### Lamin A/C Participate in Mechanosignaling Defining Cell Differentiation

Currently, the role of lamin A/C in mechanosignaling is considered to be essential for regulating vital processes in the cells including migration, homeostasis, growth, and differentiation (Martino et al., 2018; Donnaloja et al., 2020). Mechanosignaling is the cell’s ability to modulate the mechanical signals into a biological response by acting on several cell functions above. In this case, lamin A/C serve as mechanosensor, receiving external stimuli from the extracellular matrix (ECM), and then transforming them into internal biological responses. Thus, lamins are mediators, helping the cells to adapt to a changing microenvironment (Isermann and Lammerding, 2013; Guilluy et al., 2014; Osmanagic-Myers et al., 2015; Martino et al., 2018).

The first piece of knowledge about the fact that external mechanical force could lead to cell response resulting in nucleus deformation was obtained by Maniotis et al. (1997) in the 1990s. Since then, knowledge has been accumulating about the mechanisms underlying the signal transduction from the exogenous environment through the cytoplasm to the nucleus.

**FIGURE 6:**
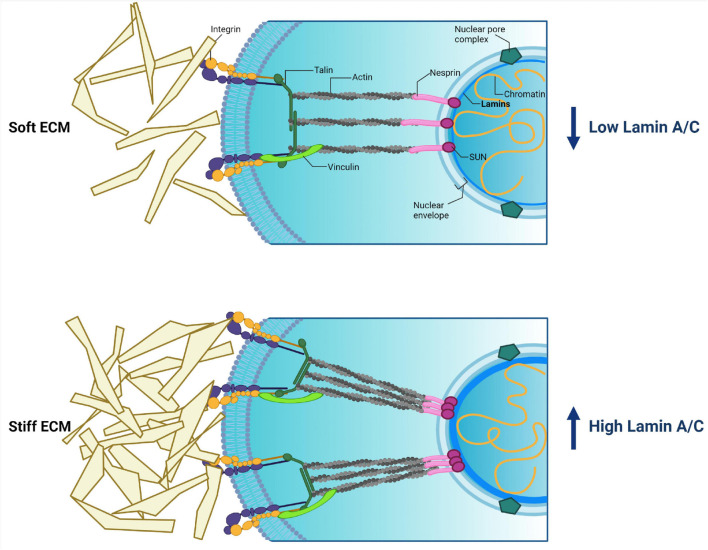
Cytoplasmic and nuclear components involved in mechanotransduction and their relations. External stimuli coming from extracellular matrix (ECM) pass to cytoplasmic membrane integrins. Then the signal transmits though focal adhesion proteins (talin, a-actinin, vinculin) toward actin. Actin directly binds with the LINC complex (nesprin + SUN + lamins), resulting in the signal being transmitted into the nucleus. The force of external stimuli affects lamin A/C production, thereby driving nucleus stiffness and protecting the genome. Created with BioRender.com.

ECM includes many components (proteins, glycosaminoglycans, and proteoglycans) that impact the cell surface in a specific manner. The most common of them are collagen, laminin, and fibronectin (Figure 6). The ECM composition is unique for a given tissue and could be changed in response to alteration of the environment, especially in the case of a disease (Bonnans et al., 2014). The mechanical signal from ECM is transmitted to membrane-bound integrins that perform a sensor role. Integrins mediate the transformation of mechanical stimuli into biochemical signals. Interestingly, depending on the quantity and type of integrins, cells can react in a different way (Israeli-Rosenberg et al., 2014). Through the accumulation of proteins termed focal adhesion complex (FAC), integrins are associated with the cytoskeleton. FAC proteins, such as talin, a-actinin, and vinculin, define the strength of interaction between integrins and filamentous actin (F-actin), which is a general cytoskeleton component (Chin et al., 2019). Then the signal is translocated *via* the LINC complex to nuclear lamins, the main sensors of mechanotransduction (Figure 6).

The importance of lamin A/C in mechanotransduction was confirmed in studies where cells lacking lamin A/C or expressing *LMNA* mutants were unable to directly transmit forces to the nucleus (Poh et al., 2012). In addition, signaling cascades, such as ROCK, Src, and ERK are known to be implicated in the mechanotransduction process (Osmanagic-Myers et al., 2015).

Despite the identification of a spectrum of molecular components involved in mechanotransduction, it remains completely unknown how these components act and adapt to each other to affect cellular functions and stem cell fate. The differentiation process is believed to be mechanosensitive, and cell fate could be determined by type and physical force of external stimuli. Current proposed model could be as follows. During cell differentiation A-type lamins get information about the changing microenvironment from nearby cells and ECM through the cytoskeleton. This leads to a rearranging meshwork and chromatin structures, or urges conformational changes in nuclear proteins such as transcription factors and components of signaling pathways. It is supposed that these conversions lead to chromatin segments’ translocation away from or to the lamina, resulting in activation/repression of differentiation-related genes (Swift et al., 2013; Alcorta-Sevillano et al., 2020). First of all, this could be determined by the physical properties of the tissues. Some researchers have revealed correlations between substrate stiffness and gene transcription intensity of lamin A/C in a tissue-specific manner. For instance, Heo et al. (2016) have demonstrated that low external stimuli promote mesenchymal stem cell (MSC) to adipogenic differentiation associated with inhibited lamin A/C production. Other authors revealed that medium force stimuli induce MSC to differentiate into myocytes’ direction, which is accompanied by elevation of lamin A/C expression (Engler et al., 2006; Swift et al., 2013). In addition, high lamin A/C expression level of hard tissues (such as bone) stabilizes the nucleus against mechanical stress. At the same time, soft tissues, such as fat, are characterized by a low expression level of lamin A/C. It has been demonstrated that lamin A/C knockdown enhances mesenchymal stem cell differentiation on a soft matrix, which contributed to fat phenotype development. In contrast, lamin A/C overexpression enhances cell differentiation on a stiff matrix toward a bone phenotype (Swift et al., 2013; Alcorta-Sevillano et al., 2020). In addition, lamin A/C overexpression leads to an inhibition of chromatin remodeling, and also to an activation of other actions such as expression of stress-related proteins implicated in cell differentiation, and transcriptional regulator YAP1 involved in cell proliferation and the suppression of apoptotic genes and Hippo pathway (Swift et al., 2013).

Thus, *via* adhesion proteins and cytoskeleton meshwork, ECM transmits information into the nucleus about the microenvironment to stabilize proper shape and stiffness of the nucleus by means of the quantity of lamins. High lamin A/C expression protects all components of the nucleus from severe forces coming from a stiff ECM, for example in a bone tissue. This mechanism reflects a mechanical theory of lamin A/C’s role in the cells (Osmanagic-Myers and Foisner, 2019; Figure 6).

Some researchers have demonstrated the importance of the Ig-domain of lamin A/C in stress-related changes in terms of lamina rearrangement. In response to stress, electrostatic interaction between the positively charged Ig-tail domain and negatively charged regions of the rod domain of a nearby lamin’s filament is disrupted, resulting in lamina reorganization (Makarov et al., 2019).

Thus, the expression level of lamin A/C determines tissue-specific differentiation of cells. In this way, mechanical signals coming from the intercellular matrix can direct lamins to proper stabilization of the genome in response to mechanical stress and tissue-specific gene expression during cell differentiation. These events are necessary to support nucleus shape and prevent the DNA from breaking.

### Lamin A/C Regulates Chromatin Organization and Gene Expression

Genomic DNA in the eukaryote nucleus is known to be extensively packaged in chromosomes, each of which occupies a certain area termed the chromosome territory (Cremer and Cremer, 2010). According to transcriptional activity, chromatin is divided into euchromatin, which includes the majority of actively expressed genes, and heterochromatin, including transcriptionally inactive genes. Heterochromatin mostly occupies the nuclear periphery, whereas euchromatin is localized in the interior part of the nucleus. In addition, heterochromatin is sub-divided into constitutive heterochromatin, which is localized in the pericentromeric and subtelomeric regions of chromosomes, and facultative heterochromatin, localized in chromosome shoulders (Lieberman-aiden et al., 2009; Ou et al., 2017). It has been shown that heterochromatin is associated with lamin A/C forming the nuclear lamina, while euchromatin dominating in the nuclear interior is connected with a small number of nucleoplasmic lamin A/C. A-type lamins are considered to regulate the repressive state of genes included in facultative heterochromatin (Gruenbaum and Foisner, 2015; de Leeuw et al., 2018; Bitman-Lotan and Orian, 2021). This three-dimensional organization of chromatin contributes to the gene expression regulation and maintenance of silencing of heterochromatic genes.

Nowadays, the multiplicity of methods such as super-resolution microscopy (Cremer et al., 2017; Ricci et al., 2017), chromosome capture methods (Dekker et al., 2002), and chromatin immunoprecipitation (ChIP) allow deeper investigation of 3D nuclear architecture (Collas, 2010; Oldenburg and Collas, 2016). In this way direct interactions of chromatin with lamin A/C were identified using DNA adenine methyltransferase identification (DamID) (Van Steensel and Henikoff, 2000; Guelen et al., 2008) and chromatin immunoprecipitation methods (Lund et al., 2014, 2015). These regions now are broadly known as lamina-associated domains (LADs). Approximately 30–40% of the genome is occupied by LADs, which contain different gene sets in a silent state according to the particular type of cells. Moreover, it has been suggested that lamin A/C located in the nuclear interior as well as peripheral lamin A/C (as a part of lamina) are involved in gene repression (Naetar et al., 2017). Similar to heterochromatin, there are facultative and constitutive LADs (fLADs and cLADs, respectively). The set of cLADs is very identical in cells from several origins. Conversely, fLADs are unique for different cells types (Melcer and Meshorer, 2010). During several studies, it has been demonstrated that fLADs are spatially positioned in tissue-specific and embryo stage-dependent ways (Robson et al., 2016; Poleshko et al., 2017, 2019). Recent research conducted on induced pluripotent stem cells (iPSC) carrying a tissue-specific *LMNA* mutation has confirmed this fact and determined that disruption of lamin–chromatin bonds occurs in regions with specific characteristics. Using three cell types such as cardiomyocytes (iPS-CMs), adipocytes (iPS-adips), and hepatocytes (iPS-heps), obtained from iPSC with one of the two cardiac-specific *LMNA* mutations (T10I and R541C), it has been determined that LADs have cell-specific organization. Moreover, cardiac-specific *LMNA* mutations have a more destructive effect on iPS-CMs compared with iPS-adips and iPS-heps (Shah et al., 2021).

During mitosis, dividing cells undergo some nuclear events, including release of transcription factors and chromatin reorganization accompanied by rearrangement of LADs. Interestingly, these cell-type– specific changes could be reconstructed after mitosis (Shevelyov and Ulianov, 2019). The molecular mechanisms of maintenance of cell-specific orientation of LADs remain unknown. Also, it is not fully clear how chromatin is attached to nuclear lamina. Several studies aimed on identification of relations of nuclear lamins with genome in different cells’ types throughout the cell cycle. For instance, Kind et al. (2013) investigated the dynamics of interaction of LADs with nuclear lamina using DNA adenine methylation to visualize and track LADs in single human cells. Early after mitosis a stochastic character of the LADs-nuclear lamina contacts was identified and this was associated with gene repression and positively correlated with H3K9me2 histone modification (Kind et al., 2013). In another report modified DamID protocol was used to map the interaction of nuclear lamins with the genome in single human cells. Gene-poor LADs contacting with the genome constitutively in case of different cells’ types were identified. In addition, there are LADs with more variable lamin-genome interaction, that are cell-type specific. Furthermore, constitutive LADs are characterized by low gene activity and heterochromatic histone modification H3K9me (Kind et al., 2015). Recent research has shown significant results about the dynamics of LADs during interphase, in particular at the onset of G1 phase and during DNA replication. Antibody-based variant of the DamID technology (pA-DamID) allowed to map and visualize nuclear lamina–genome interactions with high temporal resolution. Obtained results showed that after mitosis lamins-genome contacts are widespread on distal regions of chromosomes. Small LADs appear to be gradually displaced from the nuclear lamina by larger LADs. In addition, lamins contacts are increased during DNA replication (van Schaik et al., 2020). Although all these findings are important for understanding principles of the spatial LADs dynamic throughout the cell cycle, yet it remains unclear how this spatial LADs architecture is brought about and which other players are involved.

Multiple interactions of the genome and lamina along large LAD regions are known to be dependent on histone post-translational modifications. Poleshko et al. (2019) have demonstrated that the H3K9me2 mark takes part in 3D spatial heterochromatin organization at the nuclear periphery, and re-associates with the forming nuclear lamina after mitosis. Besides, H3K27me3 marks, as well as CTCF binding sites, flank LADs, mediating their anchoring to the nuclear envelope (Harr et al., 2015).

In addition, apart from lamin A/C participating in chromatin organization, INM proteins can bind genome regions with nuclear lamina, resulting in gene silencing. So it has been shown that LBR is connected with the histone modification H3K9me3 through heterochromatin-binding protein 1 (HP1) (Hirano et al., 2012). Emerin is able to interact with HDAC3 by initiating its catalytic activity (Demmerle et al., 2012). The LAP2β protein plays a critical role in genome organization, gene expression and differentiation process *via* interaction with the ATP-dependent chromatin remodeling complex BAF (mammalian SWI/SNF complex) (Margalit et al., 2007). There are more examples of the involvement of INM proteins in the regulation of chromatin architecture which can be found in previous reviews (Cai et al., 2001; Zuleger et al., 2011).

**FIGURE 7:**
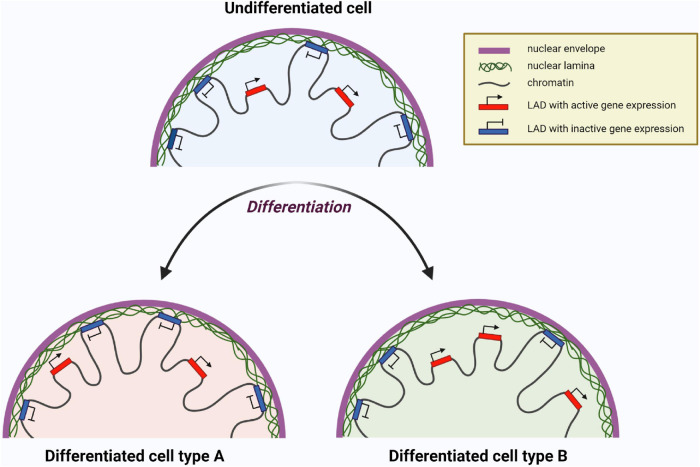
Chromatin reorganization resulting in change of gene expression profile of a cell during differentiation. Lamina-associated domains (LADs) attached to nuclear lamina are transcriptionally inactive, whereas LADs unattached to lamina are available for transcription. The set of LADs is unique for certain differentiated cells and different from undifferentiated cells. Here two hypothetical differentiated cell types are shown as ”Differentiated cell type A” and ”Differentiated cell type B” having different LADs with transcriptionally active (red) and inactive (blue) genes. Created with BioRender.com.

The processes of maintaining stem cells in a pluripotent state, as well as their decision to differentiate in a certain direction, are under regulation *via* complex intracellular programs. These programs can be realized throughout changes of the activity of transcription factors, chromatin organization reconstitutions, epigenetic regulator activity, and many other events. In this regard, it is worth noting the exclusive role of lamin A/C as a part of chromatin organization and regulation of differentiation-related gene expression, resulting in the cell’s choice of further fate and specification of an identity. During cell differentiation, spatial relocation of genomic regions toward or away from lamina occurs, as is shown in Figure 7. Thus, genes non-relevant to differentiation interact with lamina and become silent. At the same time, differentiation-related genes unattached from lamina are available for their expression, facilitating the development of a particular cell identity (Bitman-Lotan and Orian, 2021).

The active role of lamin A/C in stem cell identity and cell differentiation has been investigated in several studies. For example, during myogenesis some genes move in and out of LADs in a specific way, leading to changes in their expression state. Some of these genes encode NETs (see above). This tissue-specific NET expression is significant for selective chromosomes docking near the nuclear periphery (Robson et al., 2016). Our group has shown an impact of various *LMNA* mutations on unique expression pattern of genes during MSC differentiation (Malashicheva et al., 2015). Another study of our group has shown that tissue-specific R482L *LMNA* mutation interferes with the differentiation of human cardiac mesenchymal cells into adipocytes when the Notch pathway is activated (Perepelina et al., 2018). Using lentiviral introduction of *LMNA* mutations we have also shown that A-type lamins participate in driving the osteogenic phenotype of four cell lines of mesenchymal origin in a different way (Perepelina et al., 2019).

Besides the functions described above, A-type lamins bind to the retinoblastoma protein pRb, one of the main cell cycle regulators, and are also involved in the regulation of apoptosis and in the processes of muscle and adipogenic differentiation (Boban et al., 2010; Kennedy and Pennypacker, 2014). Lamin A/C involvement in cell differentiation is also confirmed by the direct interaction of lamin A/C with cyclin D3 in muscle cells as well as with SREBP1, an important factor of adipogenic differentiation, in pre-adipocytes (Mariappan et al., 2007). The complex of lamin A/C and emerin could also interact with α-catenin and thereby determine the onset of adipogenesis (Boban et al., 2010). In addition, A-type lamins retain factor c-Fos at the nuclear periphery, which leads to the repression of transcriptional activity of AP-1 factor, a well-known regulator of cell proliferation, differentiation, and apoptosis (Mirza et al., 2021). Thus, A-type lamins are associated with many transcriptional regulators in the nucleus and can influence gene expression by binding to these factors or by affecting the basic transcriptional complexes assembly.

The C-terminal immunoglobulin-like domain of lamin A/C directly interacts with the PCNA replication factor, which plays an important role in DNA replication (Shumaker et al., 2008; Cobb et al., 2016). In natural conditions, lamin A/C expression leads to inhibition of PCNA and dephosphorylation of Rb, which consequently inactivates transcription factors of the E2F group. This leads to the arrest of the cell cycle, suppression of DNA replication, and initiation of the differentiation process. Impaired lamin A/C expression could lead to phosphorylation of Rb by the cyclin D - cdk4/6 complex and the release of transcription factor E2F. As a result, cells do not proceed to the process of differentiation, and the apoptotic mechanisms are activated (Chen et al., 2019).

Despite many discoveries regarding the role of lamin A/C in the regulation of gene expression and chromatin organization, there is still no clear understanding of all the molecular participants in these processes. Given the complexity and distinction of each specific cell type mechanism of differentiation regulation, further studies are needed on the development mechanism of severe hereditary diseases associated with impaired tissue differentiation—laminopathies.

### Lamin A/C Cooperates With Signaling Pathways During Cell Differentiation

Aside from the lamin A/C functions discussed above, they are capable of modulating the activity of signaling molecules *via* their interaction with gene regulators, promoters, and the other components of signaling cascades in the cells. Intermolecular interactions of lamin A/C with plenty of molecular signaling components or their intermediates occur due to different post-translational modifications that lamin A/C may undergo (Maraldi et al., 2010; Gerace and Tapia, 2018). As a whole, post-translational modifications of lamin A/C can be subdivided into phosphorylation, sumoylation, farnesylation, and carboxymethylation. However, the influence of these modifications on lamin A/C cooperation mechanisms with other molecules and proteins remains largely unknown (Andrés and González, 2009; Gerbino et al., 2018).

### Wnt/β-Catenin

The Wnt/β-catenin signaling pathway plays a decisive role in the differentiation of various cells *via* regulation of the genes involved in mesenchymal tissue proliferation and differentiation. It has been shown that β-catenin (intracellular signal transducer in the Wnt/β-catenin signaling) is capable of interacting with lamin-binding protein emerin, thereby controlling the expression level of emerin in differentiated cells. Inhibition of GSK3 kinase, an important step in β-catenin activation, is required for adipogenic lineage differentiation. In contrast, GSK3-kinase activation leads to differentiation of stem cells toward the osteogenic lineage (Maraldi et al., 2011). Using knockout mice (*Lmna* −/−), Tong et al. (2011) have shown that the absence of lamin A/C synthesis leads to suppression of myogenic and osteogenic cell differentiation, which correlates with an increase of adipose tissue content and with expression of adipogenic markers, as well as with decreased activity of the Wnt/β-catenin signaling pathway. The implication of Wnt/β-catenin signaling in osteogenic differentiation promotion of MSCs was confirmed in several studies (Tong et al., 2011; Wang et al., 2017), whereas adipogenic and chondrogenic direction of differentiation was suppressed when Wnt/β-catenin was activated (Case and Rubin, 2010; Ullah et al., 2015).

### Notch Pathway

Notch signaling is a key regulator of main cellular processes including proliferation, differentiation, and apoptosis in both the adult organism and the developing embryo (Schwanbeck et al., 2011; Hori et al., 2013). The Notch pathway includes four Notch receptors (Notch1, Notch2, Notch3, Notch4), five ligands (Jag-1, Jag-2, DLL1, DLL3, DLL4), and gene regulators. Receptors and ligands are mainly transmembrane forms of proteins that ensure the interaction of neighboring cells with each other. Notch receptors undergo sequential proteolytic cleavages upon binding of their ligand, resulting in the release of Notch intracellular domain (NICD) from the cellular membrane. NICD is translocated into the nucleus, where it interacts with transcription factors, thereby activating expression of target genes (Andersson et al., 2011; Henrique and Schweisguth, 2019).

Notch is established to regulate the cell differentiation process (Bray, 2006). Moreover, the involvement of Notch signaling in Hutchinson-Gilford progeria syndrome (HGPS) has been shown (Pereira et al., 2008). HGPS is associated with expression of a truncated form of prelamin A called progerin, whose accumulation mainly leads to abnormal nuclear shape and chromatin structure. Thus, mostly mesenchymal tissues are thought to be damaged. Scaffidi and Misteli (2008) showed that the expression of progerin in human MSCs causes hyperactivation of the main targets of the Notch signaling pathway—*HEY1* and *HES1* (2008). This contributes to a change in the expression of differentiation markers: enhanced adipogenic and reduced osteogenic ones. However, changes in the chondrogenic differentiation in the cells carrying the mutation, in contrast to the wild-type *LMNA*, were not observed. As a possible mechanism, it has been suggested that the presence of progerin causes a disruption of a connection of lamin A/C with the transcription factor SKIP, an activator of genes of the Notch family, thereby increasing Notch-related gene expression inside the nucleus. In addition, Notch genes probably can directly interact with the nuclear lamina, and their regulation is associated with epigenetic modifications (Scaffidi and Misteli, 2008).

The impact of various *LMNA* mutations on the Notch pathway during differentiation of the cells of various mesenchymal origin has been reported. In our previous work, we proposed that the cooperation of lamin A/C with Notch signaling could be one of the mechanisms regulating MSC differentiation, based on the facts that tissue-specific *LMNA* mutations are able to influence the Notch signaling activity in MSCs (Bogdanova et al., 2014). Involvement of Notch signaling in adipogenic and osteogenic differentiation has been analyzed in another study by our group. A specific *LMNA* mutation (R482L), associated with Dunningan-type familial partial lipodystrophy, contributes to the impairment of adipogenic differentiation when Notch signaling is activated (Perepelina et al., 2018). One more study has revealed the opposite effect of R527C *LMNA* mutation associated with osteogenic phenotype of laminopathy on the expression level of *RUNX2* (a master gene of osteogenic differentiation) during osteogenic differentiation of mesenchymal cells, such as human cardiac mesenchymal cells and human aortic valve interstitial cells upon Notch activation. These results confirmed the fact of interaction of lamin A/C with Notch signaling (Perepelina et al., 2019). Thus, specific mutations in the *LMNA* gene are implicated in functional changes of Notch signaling during cell differentiation.

### TGF-β/Smad Pathway

There is considerable evidence that the TGF-β/Smad pathway is involved in bone abnormalities *via* contravention of the osteogenic differentiation process. Smad2 is known to interact with lamin-binding protein MAN1. Kondé et al. (2010) described in more detail this interaction *via* structural analysis, and revealed a UHM domain of MAN1 participating with Smad2-MAN1 link. Heterozygous loss-of-function mutation in the *MAN1* gene leads to bone abnormalities in humans, such as osteopoikilosis (sclerotic bone lesions) with or without manifestations of Buschke-Ollendorff syndrome, and melorheostosis (aberrant growth of new bone tissue on the surface of existing bones). These abnormal changes lead to increasing bone density and overexpression of TGF-b (Hellemans et al., 2004). It has been shown that MAN1 could be implicated in inactivation through competition with transcription factors for binding to Smad2 and Smad3, and it contributes to their dephosphorylation by phosphatase PPM1A (Bourgeois et al., 2013). In addition, lamin A/C can impact TGF-β/Smad signaling activity *via* interplay with protein phosphatase 2A (Van Berlo et al., 2005). To understand how A-type lamins facilitate functional changes of TGF-β/Smad pathway, further research is obviously needed.

### Mitogen-Activated Protein Kinase Pathway

The mitogen-activated protein kinase (MAPK) pathway regulates the cell cycle and differentiation process (Maraldi et al., 2011).

A-type lamins mediate retaining c-Fos (transcription factor that regulates key cellular processes, including differentiation) at the periphery of the nucleus. Cooperation of lamin A/C with c-Fos factor could be disrupted due to phosphorylation of c-Fos by MAPK Erk. This result suggests the participation of lamin A/C in MAPK pathway activity (Gonzàlez et al., 2008). In knockout mouse models of dilated cardiomyopathy with the *LMNA* H222P mutation in response to mechanical stress in cardiomyocytes, activation of the MAPK signaling pathway was observed, in which kinases such as ERK1/2 and JNK were involved. In addition, inhibitors of this signaling pathway were found to prevent the development of cardiomyopathies associated with a mutation in the *LMNA* gene, but did not affect the development of muscular dystrophy (Muchir et al., 2007).

Thus, A-type lamins are associated with many signaling pathways and transcriptional regulators in the nucleus and could influence gene expression by binding to these factors or by affecting the assembly of basic transcriptional complexes.

## Laminopathies as a Consequence of Mutations in the *Lmna* Gene

Laminopathies are a group of hereditary diseases caused by mutations in genes encoding (a) nuclear lamins; (b) proteins associated with post-translational modifications of lamins (such as ZMPSTE24); (c) proteins that interact with lamins (emerin, LAP2, LBR, MAN1, nesprins), and (d) proteins that make up nuclear pores (Zaremba-Czogalla et al., 2011).

**FIGURE 8:**
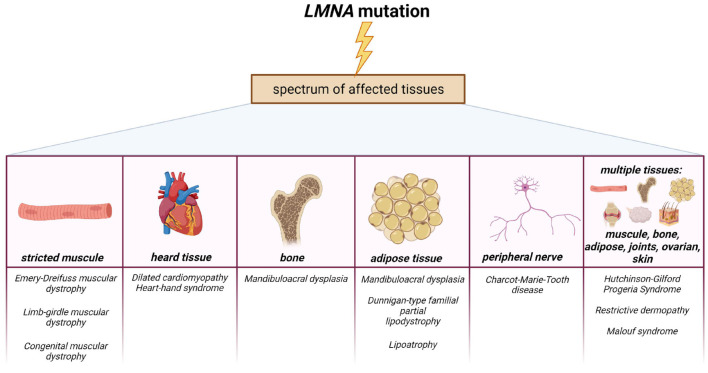
Phenotypic spectrum of laminopathies and the different tissues affected. Created with BioRender.com.

Over the past 20 years, it has been found that most laminopathies are caused by mutations in the *LMNA* gene, which encodes lamin A/C. To date, over 15 different diseases have been described, associated with 498 mutations in the *LMNA* gene as reported by UMD-LMNA, universal mutations database.^1^ Laminopathies are characterized by a wide range of clinical phenotypes, in which one type of tissue is most often affected, mainly of mesenchymal origin, for example, lipodystrophy (damage to adipose tissue), mandibuloacral dysplasia (damage to bone tissue), cardiomyopathy and muscular dystrophy (damage of the heart and skeletal muscles) (Rankin and Ellard, 2006). There are some groups of laminopathies in which different tissues are affected, resulting in overlapping or systemic phenotypes (Bertrand et al., 2011; Zaremba-Czogalla et al., 2011; Crasto and Di Pasquale, 2018; Figure 8).

Premature aging syndrome, also known as progeria, is one of the best-studied human diseases with overlapping phenotypes, in which several tissues are affected. The pathology is caused by mutations in the ZMPSTE24 gene, mutations in the *LMNA* gene, as well as by mutations in genes encoding DNA repair proteins, such as in RecQ protein-like helicases (RECQLs) and nuclear excision repair (NER) proteins and others (Navarro et al., 2006). The most famous form of progeria is Hutchinson-Guildford syndrome (Rankin and Ellard, 2006; Worman et al., 2010). This is an extremely rare autosomal dominant disease, a childhood form of progeria, characterized by changes in the skin and internal organs caused by premature aging of the body. In 2003, the mechanism of this disease development was described. A mutation in the *LMNA* gene causes the substitution of cytosine with thymine amino acid, thus forming an additional splice site in exon 11, resulting in a truncated mRNA of *LMNA* transcript. In the process of translation, an altered form of prelamin A is synthesized, in which the CaaX motif is not cleaved, and instead of the mature lamin A, the progerin protein is formed, which cannot be incorporated into the nuclear lamina resulting in disruption the scaffold of the nucleus (Gonzalo et al., 2017).

Unlike HGPS, for other diseases associated with mutations in the *LMNA* gene, the molecular mechanisms of pathogenesis are still poorly understood. Most mutations in the *LMNA* gene affect the heart or skeletal muscles. Among such diseases, Emery-Dreifuss autosomal dominant and recessive forms of muscular dystrophy (EDMD) could be distinguished (Worman, 2012). The disease was found to be associated with the R453W point mutation of the *LMNA* gene mapped to locus lq 21.2–21.3 (Favreau et al., 2004). Later, missense mutations were found, for example, G232E, Q294P, and R386K, leading to the development of EDMD (Muchir and Worman, 2007). Other diseases of the heart and skeletal muscles associated with mutations in the *LMNA* gene were soon described: dilated cardiomyopathy 1A (Fatkin et al., 1999) and limb-girdle progressive muscular dystrophy 1B (Muchir et al., 2000). EDMD, isolated dilated cardiomyopathy, and limb-girdle muscular dystrophy are characterized by overlapping clinical phenotypes and dilated cardiomyopathy associated with cardiac conduction abnormalities (Cattin et al., 2013).

Dunnigan-type familial partial lipodystrophy, also known as FPLD, is an autosomal dominant disorder characterized by a loss of hypodermic adipose tissue in the limbs and torso after puberty and excess fat deposition in the head and neck region. A total of 90% of the *LMNA* mutations in this syndrome are missense mutations located in exon 8 (Boguslavsky et al., 2006). Several such mutations have been described, for example, R482Q, R482W, G465D in exon 8, and R582H in exon 11 of the *LMNA* gene (Garg et al., 2001).

Mandibuloacral dysplasia (MAD) is a rare autosomal recessive disorder characterized by post-natal bone anomalies. MAD occurs due to point *LMNA* mutations associated with amino acid substitutions (Garg et al., 2005). Mandibuloacral dysplasia could also be caused by mutations in the ZMPSTE24 protease, involved in the processing of prelamin A to lamin A/C (Agarwal et al., 2003).

Thus, these few main examples of laminopathies demonstrate that mutations in the same *LMNA* gene could lead to the development of severe abnormalities, characterized by a wide range of clinical tissue-specific phenotypes. However, the mechanism of development of these diseases is still not fully understood.

**FIGURE 9:**
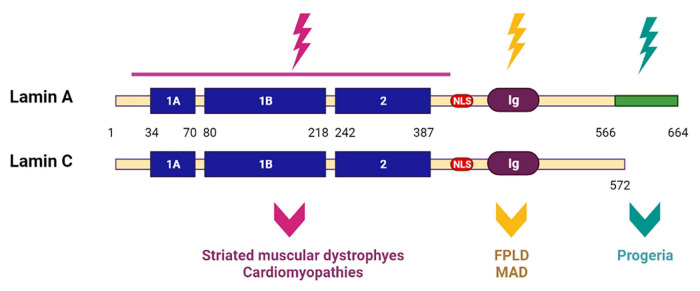
Scheme of lamin A and lamin C structures with mutated regions. *LMNA* mutations upstream of the nuclear localization signal (NLS) affect the conserved core domain. Resulting in striated muscular dystrophies and cardiomyopathies. *LMNA* mutations located downstream of NLS lead to progeroid syndromes, Dunnigan-type familial partial lipodystrophy (FPLD) and mandibuloacral dysplasia (MAD). Created with BioRender.com.

Several years ago, scientists proposed two hypotheses explaining the development of laminopathies: a structural hypothesis and a gene expression hypothesis. According to the structural hypothesis, mutations in the *LMNA* gene cause, first of all, weakening of the nuclear membrane, which makes it vulnerable to damage resulting in cell death and a replacement of differentiated tissue in specific cells. Another hypothesis is based on molecular mechanisms, and is related to the fact that A-type lamins are regulators of gene expression of some proteins, and mutations in the *LMNA* genes, therefore, disrupt their regulatory capacity and contribute to the disease development (Osmanagic-Myers and Foisner, 2019). Currently, there is evidence for both hypotheses. However, it is interesting that cluster analysis of *LMNA* mutations gives preference to one or another hypothesis depending on the localization of *LMNA* mutations associated with a particular type of laminopathies. Thus, it has been shown that mutations in the *LMNA* gene located upstream of the nuclear localization signal (NLS) affect the conserved core domain necessary for the formation and maintenance of the integrity of the nuclear cytoskeleton, while mutations located downstream interact more closely with chromatin and transcription factors (Hegele, 2005). Since the first group of mutations is mainly associated with a large group of muscular dystrophies and cardiomyopathies, scientists suggested that the causes of these diseases are, first of all, a violation of the formation of the lamina structure and mechanical defects. The second group of mutations belongs to other types of laminopathies—in particular, to progeroid syndromes, FPLD and MAD— and is most likely associated with disturbances in the interaction and regulation of important signaling pathways in the cell (Cattin et al., 2013; Figure 9).

Recently, research has mainly focused on the study of the molecular mechanisms of the development of laminopathies (Osmanagic-Myers and Foisner, 2019; Alcorta-Sevillano et al., 2020; Shah et al., 2021). The molecular mechanisms proposed by scientists include disturbances in the organization of heterochromatin, intracellular signal transduction, and in the process of autophagy, which ultimately leads to the regulation of the expression of various genes (Wong and Stewart, 2020). It is clear that iPSC-derived patient-specific models could greatly contribute to the understaning of laminopathies. Particularly, we recently generated several iPSC lines carrying disease-specific mutations in the *LMNA* gene. These iPSC lines would be a useful tool to investigate disease development of laminopathies (Perepelina et al., 2019, 2020a,b; Klauzen et al., 2020).

## Conclusion

Nuclear A-type lamins play a critical role in vital cell functions including migration, growth, homeostasis, proliferation, differentiation, and many others. Despite the fact that a broad range of studies are devoted to investigating the role of lamin A/C in the cells, all their functions are still incompletely understood and demand further investigations. A-type lamins undoubtedly play critical role in cell differentiation and determination of their identity.

To date, a large amount of data has been collected that is consistent with the hypothesis that A-type lamins define cell identity *via* the organization of chromatin architecture, epigenetic regulation and expression of differentiation related genes. Apparently, during cell differentiation, A-type lamins perform a key role in directing stem cells to a proper differentiating state. It is supposed and, for some cases experimentally shown, that for this, pluripotent or inappropriate differentiation-related genes are included in the LADs, resulting in inactivation of their expression. On the other hand, genes required for specific differentiation are activated *via* detachment of genome regions from the nuclear lamina. These genome reorganizations are cell cycle stage-dependent, and vary in the different cells from different origins (Bitman-Lotan and Orian, 2021; Shah et al., 2021).

Laminopathies are known to be associated with cell differentiation impairment. The failure of lamin A/C folding in the right way due to *LMNA* mutations leads to the disruption of integrity and function of the nuclear lamina. As a result, the interaction of chromatin with lamins can be disrupted, which leads to the essential cell and genome functions breaking, including transcription repression of the genes responsible for differentiation, and, ultimately, to the development of the disease (Scaffidi and Misteli, 2008; Melcer and Meshorer, 2010; Zhang et al., 2019). For a better understanding of the mechanisms leading to the disruption of cell differentiation, it is important to study the totality of possible changes in the cell that occur as a result of mutations in the *LMNA* gene. Currently, the most relevant studies are devoted to the epigenetic mechanisms of laminopathy development. Post-translational modifications of histones, as well as A-type lamins, are key mechanisms directing chromatin attachment to the lamina (Scaffidi and Misteli, 2008; Collas, 2010; Melcer and Meshorer, 2010; Poleshko et al., 2019; Shevelyov and Ulianov, 2019; Zhang et al., 2019). However, currently it is not completely understood what could be other mechanisms providing LADs forming.

Numerous studies have highlighted the importance of mechanical stimuli in shaping cell and tissue function including the cell differentiation process (Bonnans et al., 2014; Makarov et al., 2019; Donnaloja et al., 2020). Mechanotransduction mediates the link between ECM and components of the cell, including cytoskeleton members and A-type lamins. Their relations make it possible to transform internal stimuli into biological response (Bonnans et al., 2014; Osmanagic-Myers et al., 2015; Heo et al., 2018). Although knowledge about regulation of cytoskeleton dynamics has already been obtained, we lack a general idea about the mechanisms by which tension, propagated through the cytoskeleton, regulates mechanical signal transition. Moreover, the complete list of members involved in the mechanosignaling process is not known.

Multiple examples demonstrate that signaling pathways are implicated in relations with A-type lamins, resulting in participation in differentiating process (Parnaik, 2008; Andrés and González, 2009; Maraldi et al., 2010, 2011; Gerbino et al., 2018). Here we have discussed the main signaling pathways taking part in lamin-mediated regulation of cell fate determination, but it is not a complete list of them. An important goal for future research will be to elaborate strategies aimed at unraveling the interactions among different signaling pathways and nuclear lamins. The study of particular cell lines and cellular pathways and a detailed description of an individual molecular portrait of each cell line should explain in more details some particular features of lamin A/C action in directing cellular differentiation.

Increasing our understanding of the functional consequences of mutations in the *LMNA* gene is certainly important. For this, novel investigations using a broad range of cell types and origins are needed, since this indeed could reflect the tissue-specific features of laminopathies. Finally, a further challenge for the future will be to apply existing knowledge to the therapy of severe hereditary diseases—laminopathies.

## Author Contributions

AM supervised the work, analyzed literature data, discussed and wrote the manuscript, and acquired funding. KP analyzed literature data, designed the figures, discussed, and drafted the manuscript. Both authors contributed to the article and approved the submitted version.

## Conflict of Interest

The authors declare that the research was conducted in the absence of any commercial or financial relationships that could be construed as a potential conflict of interest.

## Publisher’s Note

All claims expressed in this article are solely those of the authors and do not necessarily represent those of their affiliated organizations, or those of the publisher, the editors and the reviewers. Any product that may be evaluated in this article, or claim that may be made by its manufacturer, is not guaranteed or endorsed by the publisher.

## Footnotes

1
http://www.umd.be/LMNA/

## Abbreviations

BAF: barrier to autointegration factor
CaaX: motive required for prelamin A processing, where C is cysteine, a – an aliphatic amino acid and X can be any amino acid
CDK: cyclin-dependent kinase
cLADs: constitutive lamina-associated domains
CTCF: CCCTC binding factor
DamID: DNA adenine methyltransferase identification
ECM: extracellular matrix
EDMD: Emery-Dreifuss muscular dystrophy
FAC: focal adhesion complex
F-actin: filamentous actin
fLADs: facultative lamina-associated domains
FPLD: Dunnigan-type familial partial lipodystrophy
FTase: farnesyltransferase enzyme
ICMT: isoprenylcysteine carboxyl methyltransferase
Ifs: intermediate filaments
INM: inner nuclear membrane
iPSC: indused pluripotent stem cell
LADs: lamina-associated domains
LAP: lamina-associated polypeptide
LBR: LMNB receptor
LINC: linker of nucleoskeleton and cytoskeleton complex
MAD: Mandibuloacral dysplasia
MSC: mesenchymal stem cell
MTs: microtubules
NETs: nuclear envelope transmembrane proteins
NLS: nuclear localization signal
NPC: nuclear pore complex
ONM: outer nuclear membrane
PNS: perinuclear space (space between ONM and INM)
pRb: retinoblastoma protein
Rce1: RAS converting enzyme 1
Zmpste24: zinc metalloprotease.
